# *Plasmodium falciparum *isolates from Angola show the S_tct_VMNT haplotype in the *pfcrt *gene

**DOI:** 10.1186/1475-2875-9-174

**Published:** 2010-06-18

**Authors:** Bianca E Gama, Guilhermina AL Pereira-Carvalho, Florbela JI Lutucuta Kosi, Natália K Almeida de Oliveira, Filomeno Fortes, Philip J Rosenthal, Cláudio T Daniel-Ribeiro, Maria de Fátima Ferreira-da-Cruz

**Affiliations:** 1 Laboratory of Malaria Research, Instituto Oswaldo Cruz, Fiocruz, Rio de Janeiro, Brazil; 2 Health Progress and Investigation Network of the Portuguese-Speaking Countries Community (RIDES/CPLP), Centro de Malária e Doenças Tropicais, Instituto de Higiene e Medicina Tropical, Universidade Nova de Lisboa, Lisboa, Portugal; 3 National Institute of Public Health, Luanda, Angola; 4 Department of Medicine, San Francisco General Hospital, University of California San Francisco, San Francisco, USA

## Abstract

**Background:**

Effective treatment remains a mainstay of malaria control, but it is unfortunately strongly compromised by drug resistance, particularly in *Plasmodium falciparum*, the most important human malaria parasite. Although *P. falciparum *chemoresistance is well recognized all over the world, limited data are available on the distribution and prevalence of *pfcrt *and *pfmdr1 *haplotypes that mediate resistance to commonly used drugs and that show distinct geographic differences.

**Methods:**

*Plasmodium falciparum*-infected blood samples collected in 2007 at four municipalities of Luanda, Angola, were genotyped using PCR and direct DNA sequencing. Single nucleotide polymorphisms in the *P. falciparum pfcrt *and *pfmdr1 *genes were assessed and haplotype prevalences were determined.

**Results and Discussion:**

The most prevalent *pfcrt *haplotype was **S**_tct_VMN**T **(representing amino acids at codons 72-76). This result was unexpected, since the **S**_tct_VMN**T **haplotype has previously been seen mainly in parasites from South America and India. The CV**IET**, CVMN**T **and CV**I**N**T **drug-resistance haplotypes were also found, and one previously undescribed haplotype (CVM**DT**) was detected. Regarding *pfmdr1*, the most prevalent haplotype was **Y**EYSNVD (representing amino acids at codons 86, 130, 184, 1034, 1042, 1109 and 1246). Wild haplotypes for *pfcrt *and *pfmdr1 *were uncommon; 3% of field isolates harbored wild type *pfcrt *(CVMNK), whereas 21% had wild type *pfmdr1 *(NEYSNVD). The observed predominance of the **S**_tct_VMN**T **haplotype in Angola could be a result of frequent travel between Brazil and Angola citizens in the context of selective pressure of heavy CQ use.

**Conclusions:**

The high prevalence of the *pfcrt ***S**VMN**T **haplotype and the *pfmdr1 *86**Y **mutation confirm high-level chloroquine resistance and might suggest reduced efficacy of amodiaquine in Angola. Further studies must be encouraged to examine the *in vitro *sensitivity of *pfcrt ***S**VMN**T **parasites to artesunate and amodiaquine for better conclusive data.

## Background

More than 125 years after the discovery of the causative parasites, malaria continues to be one of the most important infectious diseases in the world. According to WHO, 3.3 billion people are at risk, and about 250 million malaria cases occur annually, causing nearly one million deaths, mostly in children below five years of age. Overall, 109 countries are endemic for malaria; 45 of these are in Africa [1]. While work continues toward a malaria vaccine, prompt diagnosis and effective treatment remain, in addition to vector control measures, mainstays of malaria control [2]. Unfortunately, disease control is strongly compromised by the emergence of resistance to commonly used drugs [3]. Of particular concern is resistance in *Plasmodium falciparum*, the most virulent human malaria parasite [4].

Important advances in the understanding of mechanisms of anti-malarial drug resistance have been made in recent years [5]. Resistance to chloroquine (CQ), which was previously the most widely used anti-malarial, is mainly conferred by single-nucleotide polymorphisms (SNPs) in *pfcrt*, a putative transmembrane transporter localized in the parasite digestive vacuole membrane [6]. In particular, the *pfcrt *K76**T **mutation plays a central role in mediating resistance to CQ, and it is considered a reliable molecular marker of resistance [7,8]. Polymorphisms in another gene, *pfmdr1*, are associated with modulation of parasite susceptibility to a number of drugs, including CQ and amodiaquine [9].

Regarding the *pfcrt *gene, a haplotype defined by the K76**T **codon and adjacent amino acids (numbers 72-75) has been used to type malaria parasites. Three main *pfcrt *haplotypes have been identified [10]: CVMNK among CQ-sensitive isolates from all geographic regions, CV**IET **among CQ-resistant isolates from Southeast Asia and Africa, and **S**VMN**T **among CQ-resistant isolates from South America (type **S**_tct_VMN**T**; the subscript represents codon usage) and some countries of Asia (type **S**_agt_VMN**T**) [11-15].

Although *P. falciparum *chemoresistance has been described in most malarious regions [5], limited data are available on the distribution and prevalence of *pfcrt *and *pfmdr1 *haplotypes. It was investigated the prevalence of mutations in these two genes in *P. falciparum *from Angola, where only two such molecular studies have been published previously [16,17].

## Methods

### Study site and population

Clinical samples were collected in 2007 at Luanda, the capital of Angola, from individuals living in the municipalities of Sambizanga, Imgombotas, Cazenga and Viana. Luanda has a population of approximately 4.5 million and is considered a meso-endemic malaria area, according to parasitological surveys in children < 5 years and number of malaria deaths at local hospitals. Malaria is present throughout the year, with a marked increase after rains peak during April and May [18]. The treatment policy in Angola for uncomplicated falciparum malaria in absence of first trimester of pregnancy included artemether + lumefantrine or alternatively artesunate + amodiaquine.

Patients diagnosed with falciparum malaria based on Giemsa-stained thick smear were included in this study, after obtaining informed consent. Inclusion criteria were individuals with age ≥ 18 years, uncomplicated malaria, monoinfection with *P. falciparum *and unaware of first trimester pregnancy. The study was approved by the National Institute of Public Health/Angola Ethics Research Committee with concurrence from the Instituto Oswaldo Cruz/Fiocruz.

### Study procedures

After enrollment, 5 ml of blood was collected by venipuncture and placed into ethylenediaminetetraacetic acid (EDTA) vacutainer tubes (Becton Dickinson). The samples were centrifuged (350 *g*, 10 minutes), pellets were frozen with an equal volume of cryopreservation solution (0.9% sodium chloride, 4.2% sorbitol and 28% glycerol) and transported frozen to the Instituto Oswaldo Cruz (Fiocruz), Rio de Janeiro, Brazil.

### DNA preparation

Blood samples were thawed, and 1 ml was used for DNA extraction with the QIAamp midi kit, as described by the manufacturer (Qiagen). Samples were resuspended in a final volume of 50 μl.

### Nested PCR for *pfcrt *amplification

The *pfcrt *amplification protocol was described elsewhere [19]. Briefly, 5 μl of DNA solution was added to a 45 μl mixture containing 10 pmol of primers CRTP1 (5' CCG TTA ATA ATA AAT ACA CGC AG 3') and CRTP2 (5' CGG ATG TTA CAA AAC TAT AGT TAC C 3'). In a subsequent PCR, 5 μl of the initial PCR product was mixed with 10 pmol of primers CRTD1 (5' TGT GCT CAT GTG TTT AAA CTT 3') and CRTD2 (5' CAA AAC TAT AGT TAC CAA TTT TG 3'), to amplify a 134 bp fragment comprising the pfcrt SNPs C72**S**, M74**I**, N75**E/D **and K76**T**. PCR reactions were run twice with the GeneAmp PCR System 9700 (Applied Biosystems). DNA positive and negative controls were also used in each round of amplification. The positive control consisted of DNA from blood samples from patients diagnosed with falciparum malaria based on Giemsa-stained thick smear and the negative control consisted of DNA from blood samples of individuals without history of visiting endemic areas as well as a blank tube without DNA.

### Single PCRs for *pfmdr1 *amplification

*Pfmdr1 *gene analysis was performed with two PCR reactions that were made two times using 3 μl of DNA solution in a 47 μl mixture containing 2.5 mM of MgCl_2_, 250 μM of dNTPs, 2.5 units of Amplitaq Gold^® ^DNA polymerase (Applied Biosystems), and 10 pmol of primers MDR1STF (5' CCG TTT AAA TGT TTA CCT GCA C 3') and MDR1STR (5' TTG CAA CAG TTC TTA TTC CCA 3') to amplify a 604 bp region comprising SNPs N86**Y**, E130**K **and Y184**F**; or primers MDR1ENDF (5' GCG GAG TTT TGC ATT TAG TTC 3') and MDR1ENDR (5' CAA TGT TGC ATC TTC TCT TCC A 3') to amplify a 935 bp region comprising SNPs S1034**C**, N1042**D**, V1109**I **and D1246**Y**. The first reaction was incubated at 95°C for 10 minutes, followed by 45 cycles (94°C/1 minute, 58°C/1 minute and 72°C/1 minute) plus one final incubation at 72°C for 10 minutes. The second reaction was incubated at 95°C for 10 minutes, followed by 45 cycles (94°C/1 minute and 30 seconds, 60°C/1 minute and 30 seconds and 72°C/1 minute and 30 seconds) and a final incubation at 72°C for 10 minutes. As for *pfcrt *PCR, it was also used DNA positive and negative controls in each PCR.

### PCR analysis and product purification

PCR products were separated by 2% agarose-gel electrophoresis. Products were purified through the Wizard SV Gel and PCR Clean-Up System (Promega), according to the manufacturer's instructions.

### Sequencing

DNA sequencing was performed using *pfcrt *nested or *pfmdr1 *single PCR primers plus the purified product according to Big Dye^® ^Terminator Cycle Sequencing Ready Reaction version 3.1 instructions (Applied Biosystems). Sequences were read using an ABI PRISM DNA Analyzer 3730 (Applied Biosystems) from the Genomic Platform/PDTIS/Fiocruz [20].

### Sequence analysis

The forward and reverse sequences were analysed using the free software Bioedit Sequence Alignment Editor version 7.0.5.2.

## Results

DNA blood samples from 114 patients with uncomplicated falciparum malaria were tested. The majority of samples were composed by adults with 21 to 30 years and their parasitemia range was 500-100,000 parasites/μl. We detected 102 PCR-positive samples for *pfcrt *(32 from Sambizanga, 42 from Ingombotas, 21 from Cazenga and 7 from Viana) and 28 samples for both *pfmdr1 *PCR amplifications (5 from Sambizanga, 13 from Ingombotas, 6 from Cazenga and 4 from Viana). Frequent failures to amplify *pfmdr1 *might be attributed to primer limitations due to polymorphisms in target sequences; for all these samples fragments were amplified using other primers developed for diagnosis, confirming the presence of parasite DNA in the samples [21].

Mixed infections with two different *pfcrt *genotypes were common (Table 1). The most prevalent haplotype for *pfcrt *was **S**_tct_VMN**T **(58/102). This result was unexpected, since the **S**_tct_VMN**T **haplotype has previously been seen mainly in parasites from South America and India [22,23]. The CV**IET**, CVMN**T **and CV**I**N**T **drug-resistance haplotypes were also found, and one previously undescribed haplotype (CVM**DT**) was detected. Regarding *pfmdr1*, the most prevalent haplotype was **Y**EYSNVD (17/28) (representing amino acids at positions 86, 130, 184, 1034, 1042, 1109 and 1246) with wild type sequence except for the 86 codon (Table 2). No significant difference between the four municipalities was observed for both genes.

**Table 1:** Haplotypes for *pfcrt *identified in Angola.

Haplotypes	n (%)	Number of mutated positions	95% CI
**Single-type** **(n = 83)**			

**CVIET**	13 (12.7)	3	6.28 - 19.22

**S_tct_VMNT**	58 (56.9)	2	47.25 - 66.47

**CVMNT**	4 (3.9)	1	0.15 - 7.69

**CVMNK**	3 (2.9)	0	0 - 6.22

**CVINT**	2 (2)	2	0 - 4.65

**CVMDT**	3 (2.9)	2	0 - 6.22

**Mixed-type (n = 19)**			

**C/S V M/I NT**	3 (2.9)	3	0 - 6.22

**C/S V M/I N/D T**	2 (2)	4	0 - 4.65

**C/S V M/I N/E T**	1 (1)	4	0 - 2.89

**C/S VIET**	1 (1)	4	0 - 2.89

**C/S VMNT**	11 (10.8)	2	4.76 - 16.8

**CVM N/D T**	1 (1)	2	0 - 2.89

The haplotypes were constructed considering codon positions 72-76. The 95% confidence interval was calculated by Dimension Research calculator (http://www.dimensionresearch.com/resources/resources_overview.html).

**Table 2:** Prevalence of *pfmdr1 *haplotypes in Angola.

Haplotypes	n (%)	Number of mutated positions	95% CI
**Single-type** **(n = 27)**			

**YEYSNVD**	17 (60.7)	1	42.62 - 78.8

**NEFSNVD**	3 (10.7)	1	0 - 22.16

**YEFSNVD**	1 (3.6)	2	0 - 10.44

**NEYSNVD**	6 (21.4)	0	6.23 - 36.63

**Mixed-type (n = 1)**			

**N/Y EFSNVD**	1 (3.6)	2	0 - 10.44

The haplotypes were constructed considering codon positions 86, 130, 184, 1034, 1042, 1109 and 1246. The 95% confidence interval was calculated by Dimension Research calculator (http://www.dimensionresearch.com/resources/resources_overview.html).

Wild haplotypes for *pfcrt *and *pfmdr1 *were uncommon; 3% (3/102) of field isolates harbored wild type *pfcrt *(CVMNK), whereas 21% (6/28) had wild type *pfmdr1 *(NEYSNVD). The co-analysis of *pfcrt *and *pfmdr1 *genes (n = 28) revealed some prevalent *pfcrt *and *pfmdr1 *associations: *pfcrt *CV**IET **coexisted with *pfmdr1 ***Y**EYSNVD in 29% of samples, **S**_tct_VMN**T **with **Y**EYSNVD in 14% of samples and C**VIET **with NEYSNVD in 11% of samples. Only one sample, from Imgombotas, was entirely wild type in the *pfcrt *and *pfmdr1 *genes, and three samples, from Cazenga and Imgombotas, harbored only one mutation, this at *pfmdr1 *codon 184.

## Discussion

In this study, the *pfcrt *and *pfmdr1 *haplotypes of *P. falciparum *from Angola (Luanda) were characterized. Well-known as well as unusual *pfcrt *haplotypes were identified: CVMNK representing the sensitive haplotype, **S**_tct_VMN**T**, CVMN**T**, CV**I**N**T **and CV**IET **representing previously described drug-resistance-mediating haplotypes, and CVM**DT**, a previously undescribed haplotype. These results offer insight into the spread of drug resistance in malaria parasites.

CQ-resistant *P. falciparum *parasites were first detected in the 1950 s on the Thailand-Cambodia border and in South America [24]. In Africa, resistant parasites are thought to have originated from Southeast-Asian isolates that spread progressively in the 1970 s through the Indian subcontinent and then to East Africa. Different origins of chloroquine resistance are supported by the existence of different haplotypes in different areas, notably CVMNK among CQ-sensitive isolates from all regions, and, among resistance-mediating haplotypes CV**IET **in Southeast Asia and Africa, **S**_tct_VMN**T **in South America, and **S**_agt_VMN**T **in some countries of Asia [11-15].

Surprisingly, in this study the most prevalent *pfcrt *haplotype was **S**_tct_VMN**T**. In prior studies, this **S**_tct_VMN**T **allele was the predominant haplotype in Brazil [25], and it has also been described in Guyana [26,27] and Peru [28]. It has been reported only once in Africa, in 4% of *P. falciparum *samples from Ghana [26]. In addition, an **S**VMN**T **haplotype with different codon preference (**S**_agt_VMN**T**) was seen in 19% of samples from Tanzania [29, personal communication, Michael Alifrangis]. The observed predominance of the **S**_tct_VMN**T **haplotype in Angola could be a result of frequent travels between Brazilian and Angolan citizens by means of trade, business and educational purposes, in the context of the selective pressure of heavy use of CQ. It is important to remark that Angola and Brazil have a singular relationship towards each other. Since 2000, commerce between these two countries was established and it is now flourishing. With trade enhancement, the presence of Brazilian companies in Angola has grown and consequently, short or long-terms travels from Brazil to Angola and vice-versa have also increased. An analysis of neutral microsatellite markers could help to explain if the high prevalence of the **S**_tct_VMN**T **haplotype in Angola is related to a Brazilian geographical allelic exchange or to a novel selection of resistance in Angola [26,30]. Likewise, the *pfcrt *C/**S **V**IET **mixed haplotype seen in Angola could give rise to the **S**V**IET **haplotype previously identified in the Democratic Republic of Congo [31]. Similar to potential transfer from Brazil to Angola, the presence of this haplotype in Angola might be due to traffic between the Democratic Republic of Congo and Angola. In fact, there is an expressive non-controlled migration from Congo to Angola mainly to find work at diamond mines and in a lesser extend from Angola to Congo to visit family members that were moved to Congo due to colonial Portuguese war.

The analysis of *pfmdr1 *haplotypes in Angola revealed the **Y**EYSNVD and NEYSNVD haplotypes, which were already observed in isolates from other African countries [26] and were also detected in Asia [32]. Although previous studies performed with Angolan samples only reported 2 mutations - 86**Y **for *pfmdr1 *gene and 76**T **for *pfcrt *gene - the frequencies informed for both genes are in accordance with our findings [16,17].

It is important to notice that for the *pfmdr1 *gene, associations of polymorphisms with CQ sensitivity are less straightforward than for *pfcrt*. Mutations in *pfmdr1 *also impact on responses to other drugs. Indeed, the same mutations that apparently decrease sensitivity to CQ, amodiaquine, and quinine may increase sensitivity to some other drugs, including mefloquine and halofantrine [33]. Considering this complexity, the selective pressures that led to common *pfmdr1 *haplotypes in Angola are uncertain.

Of interest, *pfcrt *and *pfmdr1 *haplotypes also impact on sensitivity to amodiaquine. In particular, the **S**_agt_VMN**T **haplotype has been associated with decreased amodiaquine sensitivity compared to other haplotypes also containing the K76**T **mutation [12,26,34], and the *pfmdr1 *N86**Y **mutation mediated decreased *in vitro *sensitivity to amodiaquine [33], and was selected by prior amodiaquine therapy [35]. In this context, the 57% prevalence of the **S**_tct_VMN**T **haplotype and 66% prevalence of *pfmdr1 *86**Y **might suggest widespread resistance to amodiaquine in Angola.

## Conclusions

The high prevalence of the *pfcrt ***S**VMN**T **haplotype and the *pfmdr1 *86**Y **SNP confirm high-level chloroquine resistance and might suggest reduced efficacy of amodiaquine in Angola. Further *in vitro *studies must be conducted to evaluate the influence of *pfcrt ***S**VMN**T **haplotype to artesunate and amodiaquine sensitivity, for better conclusive data.

## Competing interests

The authors declare that they have no competing interests.

## Authors' contributions

BEG participated in the design of the study, carried out the molecular analysis and drafted the manuscript; GALPC and FJILK were the responsible for blood samples collection; NKAO performed the PCR assays; FF helped in study design and field facilities; PJR and CTDR helped in the design of the study and reviewed the manuscript; MFFC conceived the study, coordinated its design, and finalized the manuscript. All authors have read and approved the final text.
